# Advances in Gait Alterations and Rehabilitation After Anterior Cruciate Ligament Reconstruction: Biomechanics and Emerging Technologies

**DOI:** 10.1111/os.70261

**Published:** 2026-03-03

**Authors:** TszLeung Yu, HengYu Liao, ZhenQiang Huo, ZhiYu Huang, JieRuo Li, XinYu Fang

**Affiliations:** ^1^ Department of Sports Medicine Center The First Affiliated Hospital of Jinan University Guangzhou China; ^2^ Department of Orthopaedic Surgery The First Affiliated Hospital of Fujian Medical University Fuzhou China; ^3^ Department of Orthopaedic Surgery National Regional Medical Center, Binhai Campus of the First Affiliated Hospital, Fujian Medical University Fuzhou China; ^4^ Fujian Provincial Institute of Orthopedics The First Affiliated Hospital, Fujian Medical University Fuzhou China

**Keywords:** anterior cruciate ligament injuries, biomechanics, gait analysis, kinematic asymmetries, knee

## Abstract

Anterior cruciate ligament (ACL) injuries are prevalent in sports and daily life, often leading to functional instability and long‐term complications such as osteoarthritis. This literature review synthesizes advancements in postoperative gait analysis following ACL reconstruction (ACLR), focusing on biomechanical alterations, rehabilitation outcomes, and emerging technologies. Current methodologies, including three‐dimensional motion capture, force plate kinetics, surface electromyography (sEMG), wearable sensors, machine learning and artificial intelligence, reveal persistent kinematic asymmetries, and altered joint loading patterns in ACLR patients. Rehabilitation interventions, such as neuromuscular training, biofeedback, and AI‐assisted systems, show promise in restoring dynamic stability but require standardization and cost optimization. Limitations of existing studies include small sample sizes, short follow‐up periods, and methodological inconsistencies. Future research should prioritize multicenter longitudinal studies, multimodal data integration, and AI‐driven precision rehabilitation to optimize recovery and mitigate long‐term risks. This study aims to elucidate the role of gait analysis in optimizing rehabilitation protocols and mitigating long‐term complications by evaluating the strengths and limitations of existing approaches.

## Graphical abstract

This article reviews the research advances in anterior cruciate ligament reconstruction. By evaluating the strengths and limitations of current methodologies, it elucidates the role of gait analysis in optimizing rehabilitation protocols and mitigating long‐term complications.

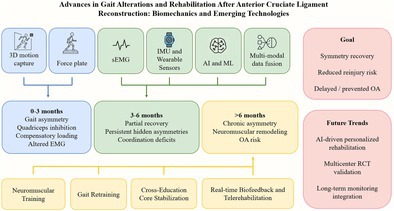

## 1 Introduction

Anterior cruciate ligament (ACL) injury is a common type of knee joint injury in sports and daily life, and anterior cruciate ligament reconstruction (ACLR) is the primary treatment for ACL injuries. Sudden stops, jumps, and directional changes often subject the knee joint to significant loads, making ACL injuries highly likely. ACL damage causes not only functional knee instability but also significantly increases the risk of meniscal injuries, cartilage degeneration, and long‐term osteoarthritis (OA). In Germany, the incidence of ACL injuries is 46 per 100,000 people [1], while in Australia, this figure is projected to reach 77.2 per 100,000 by 2030–2031 [2]. ACL injuries not only affect physical health and quality of life but also result in economic losses [3]. Studies have shown that ACL injuries can impact athletes' careers [4, 5].

In recent years, research on ACL injuries has garnered widespread attention, encompassing investigations into injury mechanisms, surgical interventions, rehabilitation strategies, and their impact on gait. Numerous studies indicate that ACL injuries compromise knee joint stability, alter movement patterns, and elevate the risk of long‐term complications such as OA. This review aims to synthesize advancements in gait analysis research following ACLR. Current studies employ diverse methodologies, including three‐dimensional motion capture, force plate kinetics, surface electromyography (sEMG), and wearable sensor technologies.

Gait represents an integrative expression of neuromuscular control, joint stability, and biomechanical loading, and is therefore a sensitive indicator of knee function following ACL injury and reconstruction.

From a clinical perspective, gait analysis can help (1) identify residual biomechanical deficits, (2) detect maladaptive compensatory strategies at an early stage, (3) stratify patients at higher risk for reinjury or degenerative changes, and (4) establish reference indicators to guide individualized rehabilitation progression and return‐to‐sport decision‐making.

While these tools provide valuable insights into lower limb biomechanics, limitations such as inconsistencies in study design, small sample sizes, and short follow‐up periods hinder the generalizability of findings. The integration of artificial intelligence (AI) and machine learning in gait analysis offers promising avenues for early diagnosis and rehabilitation monitoring, though their clinical applicability remains to be explored. Therefore, the aim of this review is not merely to summarize gait analysis techniques, but to synthesize current evidence on gait alterations following ACLR, evaluate the clinical and prognostic significance of gait assessment across different postoperative phases, and discuss how emerging technologies may refine rehabilitation strategies and long‐term outcome prediction. By evaluating the strengths and limitations of existing approaches, this study seeks to elucidate the role of gait analysis in optimizing rehabilitation protocols and mitigating long‐term complications.

## 2 Literature Search Strategy and Study Quality Assessment

### 2.1 Literature Search Strategy

A comprehensive literature search was conducted to identify studies investigating gait analysis in patients with ACL injury and following ACL reconstruction. The following electronic databases were systematically searched: PubMed, CNKI. The search covered all relevant articles published up to September 2025.

The search strategy combined Medical Subject Headings (MeSH) terms and free‐text keywords, including but not limited to: “anterior cruciate ligament,” “ACL,” “ACL reconstruction,” “ACLR,” “gait analysis,” “walking biomechanics,” “kinematics,” “kinetics,” “ground reaction force,” “electromyography,” and “sEMG.” Boolean operators (“AND” and “OR”) were used to optimize sensitivity and specificity.

Reference lists of eligible articles and relevant reviews were manually screened to identify additional studies not captured in the initial database search.

### 2.2 Inclusion and Exclusion Criteria

Studies were included if they met the following criteria:

- Involved patients with ACL injury or following ACL reconstruction;
- Employed quantitative gait analysis methods (e.g., motion capture, force plates, sEMG, wearable sensors, or AI‐based gait assessment);
- Reported biomechanical, neuromuscular, or functional gait‐related outcomes;
- Were original research articles, including randomized controlled trials (RCTs), cohort studies, or case–control studies;
- Published in English.

Studies were excluded if they:

- Were case reports, conference abstracts, editorials, or expert opinions;
- No biomechanical data;
- Involved non‐human subjects;
- Lacked sufficient methodological detail or quantitative outcome measures.

### 2.3 Study Selection Process

**FIGURE 1:**
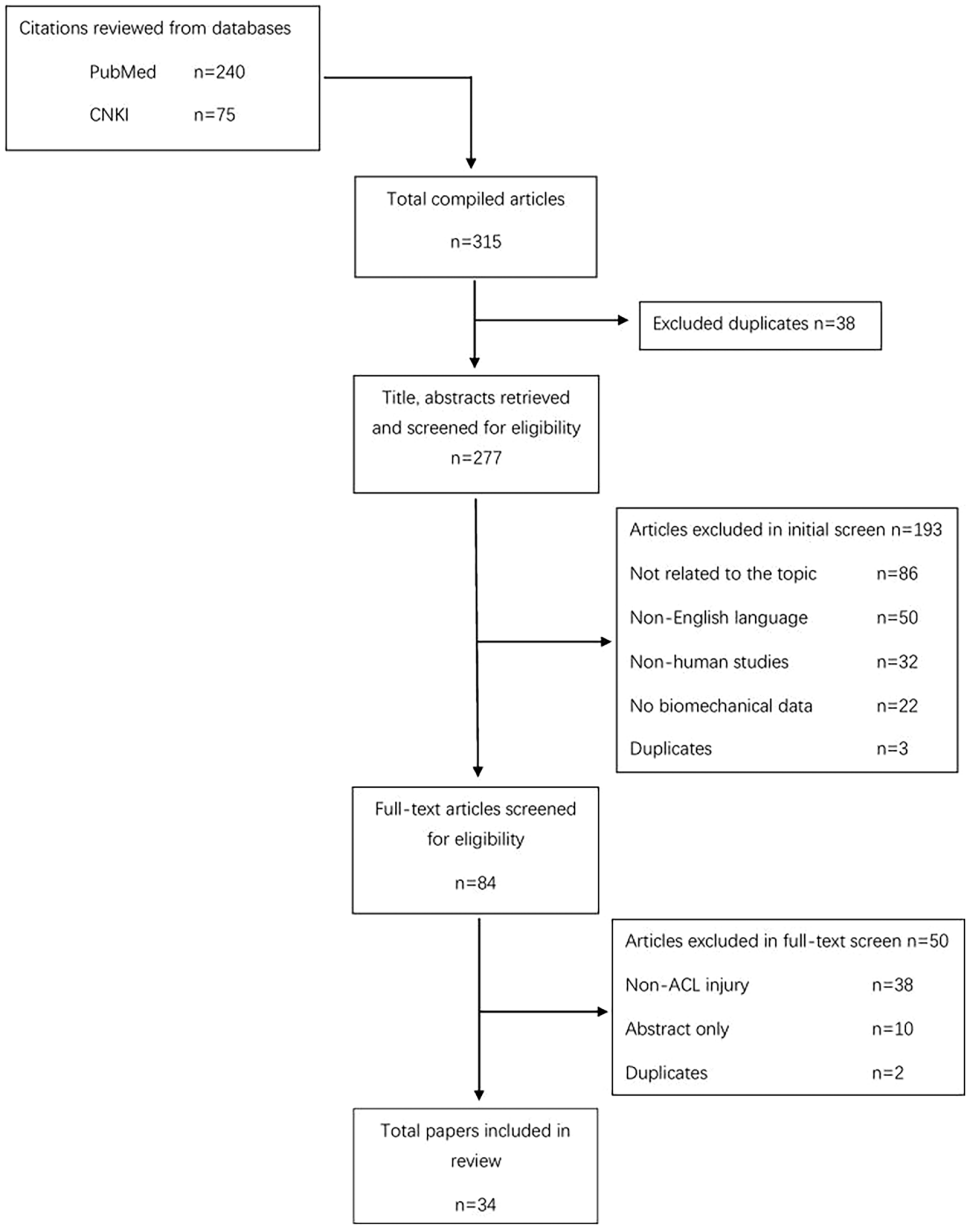
Flow chart for study screening and inclusion.

Three reviewers independently screened titles and abstracts for eligibility. Full‐text articles were subsequently assessed based on the inclusion and exclusion criteria (Figure 1).

## 3 Injury Mechanism

Current research indicates that over 70% of ACL injuries are non‐contact injuries [4], with most occurring when the knee is near full extension during landing. Non‐contact injuries typically result from sudden deceleration during directional changes or landing, whereas contact injuries are caused by knee valgus collapse [3, 6].

Non‐contact ACL injuries result from excessive stress in three anatomical planes, including anterior tibial pressure and shear force caused by quadriceps contraction, knee valgus, and impingement on the medial edge of the intercondylar spine. Under axial compression forces, an increased vertical tibial slope promotes greater posterior sliding of the lateral femoral condyle (LFC) on the lateral tibial plateau, thereby increasing ACL stress. Excessive joint stress may lead to complete ACL rupture, consistent with the bone bruising observed on MRI post‐injury [4].

In some team sports such as basketball and soccer, even minor disturbances before an athlete's injury can lead to incorrect foot landing positions, resulting in non‐contact ACL injuries. Evidence suggests that foot landing positions are modifiable and play a critical role in ACL injury prevention [3, 4].

## 4 Gait Analysis

Gait analysis is increasingly utilized in ACL injury research to assess lower limb kinematics, including motion at the hip, knee, and ankle joints. It is a discipline within biomedical fields like biomechanics, kinesiology, or podiatry. Through principles of mechanics, anatomy, and physiology, it systematically evaluates kinematics (joint angles, limb trajectories), kinetics (ground reaction forces, joint moments), and electromyographic activity during walking. Biomechanical and clinical studies show that the coordinated actions of hundreds of joints and muscles can represent each individual's unique walking pattern. Studies indicate that healthy individuals exhibit consistent gait variability, which is stable yet unique to each individual. Gait analysis can reveal the presence of certain diseases [7], providing a scientific basis for the diagnosis and treatment of ACL injuries.

### 4.1 Common Methods of Gait Analysis

1. 3D motion capture system: Tracks surface markers via infrared cameras to reconstruct three‐dimensional joint motion trajectories, serving as the “gold standard” in laboratory settings [8].
2. Force plates and kinetic analysis: Measures ground reaction forces (GRF) and their distribution to assess knee joint loading patterns. ACL‐injured patients often exhibit reduced GRF peaks during the stance phase, particularly in the early and late support phases. Christin Büttner et al. [9] used a 3D motion capture system combined with force plates and kinetic analysis, comparing the affected and unaffected limbs of ACL‐ruptured patients. They found statistically significant differences in key metrics such as vertical ground reaction force (vGRF), knee flexion angle (KFA), knee extension moment (KEM), and knee adduction moment (KAM) between ACL‐reconstructed patients and healthy controls. The 3D motion capture system effectively captures the motion of multiple joints and limbs, generating a stereoscopic model of limb movement. With its high precision, multi‐dimensional analysis, and multi‐modal data fusion capabilities, it can detect subtle motion changes and reveal the biomechanical mechanisms underlying gait abnormalities post‐ACL injury [10, 11, 12].
3. sEMG: Records the activation timing and intensity of muscles such as the quadriceps and hamstrings. Subjects undergo sEMG and gait testing during straight leg raises, normal‐speed walking, fast walking, and stair climbing. Research results indicate that sEMG and gait analysis can effectively assess the motor function of ACL‐reconstructed patients and provide guidance for developing personalized rehabilitation plans [13].
4. Inertial sensors and wearable devices: Portable accelerometers and gyroscopes enable real‐time monitoring of daily gait parameters, suitable for long‐term postoperative follow‐up. Islam et al. [7, 8] demonstrated that joint angle measurements during walking, squatting, and jumping activities using seven wearable inertial measurement units (IMUs) were comparable to proprietary software, providing accurate objective data for clinical practice.
5. Machine learning and AI: Deep learning algorithms identify abnormal gait patterns (e.g., stiff gait, pivot shift gait) to assist in early diagnosis and rehabilitation assessment [14]. Kokkotis et al. [14] applied interpretable machine learning methods to analyze walking biomechanics in ACL‐injured knees, finding that a support vector machine model achieved 94.95% diagnostic accuracy on biomechanical parameters and uncovered important parameters overlooked by traditional statistical analysis. Li Haoran et al. [10] collected running data through experiments, analyzed knee joint flexion and muscle strength using a musculoskeletal model, and ultimately constructed a multivariate linear regression model with an accuracy of 81.4%, providing objective evidence for the clinical diagnosis of ACLD. Tedesco et al. [15] used inertial sensors and multiple machine learning models to study gait patterns during athletes' directional changes, showing that healthy and post‐injury groups could be successfully distinguished with 73.07% accuracy.

Multi‐modal data fusion utilizing these techniques enables a more comprehensive analysis of the biomechanical mechanisms before and after ACL injury, thereby providing a scientific foundation for precise rehabilitation.

### 4.2 Postoperative Gait Analysis and Biomechanical Characteristics

Gait recovery after anterior cruciate ligament reconstruction is a dynamic and staged process characterized by continuous adaptive changes in biomechanical and neuromuscular aspects.

In the early, middle and late postoperative stages, patients exhibit distinct but interrelated gait asymmetries, altered joint loading, and compensatory movement strategies.

Early recovery is mainly characterized by protective unloading and quadriceps inhibition, while mid‐recovery is characterized by partial recovery of kinematics, but there is still persistent movement asymmetry.

In the late stages, although clinical recovery and permission to return to sport have been achieved, some subtle but clinically significant gait deviations often remain and are associated with reinjury risk and post‐traumatic osteoarthritis.

Importantly, evidence from randomized controlled trials and longitudinal studies suggests that rehabilitation strategies that incorporate neuromuscular training, biomechanical feedback, and symmetry‐focused interventions are superior to traditional time‐based programs in restoring gait mechanics.

#### 4.2.1 Preoperative to Early Postoperative Phase (Pre‐Surgery to 3 Months)

The first 12 weeks following ACL reconstruction represent a critical window of neuromuscular reorganization and compensatory gait adaptation. During this stage, patients typically exhibit pronounced gait asymmetries, quadriceps inhibition, and reduced joint loading, which if left unaddressed may persist chronically and predispose to secondary injuries [9, 13, 16, 17, 18].

From a biomechanical perspective, early postoperative gait is consistently associated with reduced knee flexion angle during loading response, diminished peak knee extensor moments, and altered hip‐knee‐ankle coordination patterns [13, 17]. These changes collectively indicate a cautious gait strategy that limits sagittal‐plane knee loading during stance.

Surface electromyography further supports the presence of neuromuscular inhibition in this phase, demonstrating delayed quadriceps activation, prolonged quadriceps–hamstring co‐contraction, and compensatory over‐reliance on the uninvolved limb. Such neuromuscular patterns contribute to inefficient force transmission across the knee joint and reinforce protective unloading behaviors during walking [9, 13].

Taken together, the dominant gait features in the early postoperative phase reflect a combination of mechanical protection and neural inhibition rather than strength deficiency alone. This distinction underscores the clinical importance of early interventions that target neuromuscular activation and movement quality, rather than focusing exclusively on strength restoration or time‐based progression.

#### 4.2.2 Intermediate Recovery Phase (3 to 6 Months)

The mid‐term postoperative period (3–6 months) is considered a pivotal transition phase for ACLR patients, during which initial pain and swelling subside, and patients gradually re‐engage in functional training. However, persistent neuromuscular deficits, asymmetries, and compensatory strategies are still common during this stage and may influence long‐term outcomes and re‐injury risk.

Although patients typically demonstrate improvements in gross motor control compared to the early phase, biomechanical asymmetries often remain. Multiple studies have reported persistent reductions in knee flexion angles and moments in the operated limb during mid‐stance and terminal stance, suggestive of ongoing quadriceps avoidance strategies [12, 19, 20]. Büttner et al. [9] further confirmed that joint kinetics and kinematics, although partially restored, still display bilateral discrepancies up to 6 months postoperatively. This phase is also marked by altered sagittal and frontal plane mechanics, with some patients demonstrating excessive hip or trunk compensation to offload the affected knee [20].

The sEMG data suggest ongoing alterations in muscle activation patterns at 3–6 months. Wu et al. [13] found that ACLR patients exhibit lower co‐contraction indices and delayed onset of hamstring activity during stance, which may compromise dynamic joint stability. Similarly, clustered muscle synergy analyses have identified disrupted coordination patterns in the hamstring‐quadriceps complex during jogging, reflecting incomplete neuromuscular recovery [10].

Biomechanical analyses consistently demonstrate that patients continue to exhibit reduced knee extensor moments and altered knee joint loading patterns in the operated limb during stance, even when sagittal‐plane kinematics such as knee flexion angles appear partially normalized. These findings indicate that improvements in muscle strength do not necessarily translate into symmetrical joint kinetics or effective load transmission during gait [12, 21, 22].

From a neuromuscular perspective, sEMG studies reveal persistent abnormalities in muscle activation timing and coordination during this phase. Delayed or insufficient hamstring activation and disrupted quadriceps–hamstring coupling have been reported, potentially compromising dynamic knee stability despite adequate strength recovery [10, 13]. In response, patients may adopt compensatory strategies involving increased hip or trunk contribution to reduce mechanical demand on the reconstructed knee.

Collectively, gait characteristics in the 3–6 month period reflect a dissociation between structural or strength recovery and functional biomechanical normalization. This mismatch highlights the limitation of rehabilitation strategies that rely primarily on time‐based progression or isolated strength metrics, and underscores the need for gait‐informed rehabilitation approaches that explicitly target kinetic symmetry and neuromuscular coordination before advancing activity intensity.

#### 4.2.3 Late Recovery Phase (> 6 Months)

Beyond 6 months after ACL reconstruction, many patients are considered to have achieved satisfactory clinical recovery, as reflected by restored range of motion, improved muscle strength, and clearance for return to sport. However, accumulating evidence indicates that gait biomechanics frequently remain abnormal during this late recovery phase, revealing a disconnect between clinical recovery and biomechanical normalization [9, 16, 20, 23, 24].

##### 4.2.3.1 Persistent Gait Asymmetries and Joint Loading Deviations

Multiple studies demonstrate that gait‐related asymmetries such as reduced peak knee flexion angles, decreased knee extensor moments, and abnormal anterior tibial translation persist at 6 to 12 months post‐ACLR [9, 19, 20]. Büttner et al. [9] employed bilateral waveform analyses and confirmed that sagittal and frontal plane kinetic discrepancies were still evident at one year postoperatively, especially during single‐limb tasks. These deviations are often subtle and may not be evident during routine clinical assessments or self‐selected walking speeds, but become pronounced during higher‐demand tasks such as fast walking, running, or sport‐specific maneuvers [10, 16, 24].

##### 4.2.3.2 Neuromuscular Strategies and EMG Alterations

Chronic‐phase ACLR patients exhibit persistent disruptions in muscle activation patterns. Wu et al. [13] used sEMG and gait analysis to demonstrate abnormal hamstring‐gastrocnemius coactivation even in late‐stage recovery. These findings suggest neuromuscular rewiring rather than restoration. Lai et al. [24] found that fast‐walking trials uncovered otherwise concealed asymmetries, especially in tibial rotation and sagittal loading, indicating speed‐dependent compensations. Similar velocity‐induced discrepancies were also found in ACL‐deficient individuals compared to healthy controls [16].

From a neuromuscular standpoint, sEMG studies suggest that late‐stage gait is characterized not by recovery of pre‐injury activation patterns, but by the persistence of compensatory muscle strategies, including altered quadriceps–hamstring coordination and increased reliance on proximal musculature. These neuromuscular adaptations may reduce perceived instability in the short term, yet they simultaneously perpetuate abnormal joint loading and may contribute to the development of secondary ACL injury and post‐traumatic osteoarthritis (PTOA) [19, 23, 25].

##### 4.2.3.3 Meta‐Analyses and Rehabilitation Timeframe Re‐Evaluation

Despite increased research volume, current rehabilitation timelines are predominantly time‐based and lack integration of biomechanical endpoints. Meta‐analyses reveal that a significant portion of patients continue to exhibit pathological gait mechanics beyond 12 months, and few RCTs incorporate high‐resolution motion analysis [18, 26, 27, 28]. Moreover, rehabilitation guidelines often emphasize strength and return‐to‐sport criteria, rather than joint‐level symmetry or compensatory movement minimization [18, 29, 30].

For example, Capin et al. [23] reported that patients with second ACL injuries had distinct pre‐injury gait patterns that were detectable and potentially modifiable during rehabilitation. This underscores the importance of early identification of “at‐risk” gait profiles using integrated biomechanical and machine‐learning tools.

##### 4.2.3.4 Conclusion

Collectively, the late postoperative phase highlights that time since surgery and conventional clinical benchmarks are insufficient indicators of true functional recovery. Persistent gait compensations beyond 6 months underscore the necessity of integrating biomechanical and neuromuscular gait assessment into long‐term rehabilitation and return‐to‐sport decision‐making, rather than relying solely on symptom resolution or strength‐based criteria.

## 5 Rehabilitation Interventions

### 5.1 The Shift Toward Biomechanically‐Informed Rehabilitation

Traditional time‐based ACLR rehabilitation protocols, while widely adopted, often neglect individual biomechanical recovery trajectories. Accumulating evidence reveals that gait asymmetries, altered joint loading, and neuromuscular dysfunction persist well beyond the early postoperative window even in those cleared for return to sport (RTS) [9, 13, 20, 23]. This has prompted a paradigm shift from protocolized timelines to symmetry and function‐driven rehabilitation, emphasizing personalized, biomechanically‐anchored interventions [18, 29].

Several meta‐analyses and systematic reviews have emphasized that current rehabilitation timelines may underestimate the duration required for true movement normalization. For instance, gait deviations may persist up to 12 months or more, with joint loading discrepancies being a key precursor to second ACL injury and early‐onset PTOA [23, 28].

### 5.2 Neuromuscular Training and Asymmetry Reduction

Numerous RCTs have investigated the efficacy of neuromuscular training (NMT) to restore joint mechanics. For example, Culvenor et al. [30] implemented a phase‐based NMT protocol aimed at improving proprioceptive sensitivity and reducing asymmetric loading, achieving significant improvements in frontal‐plane knee kinetics. Similarly, the use of knee extension constraint training has been shown to facilitate improved walking biomechanics at 6 months post‐ACLR by modulating interlimb loading patterns [31].

Some studies explored contralateral limb cross‐education, where strength and control improvements in the uninjured limb indirectly enhance function in the reconstructed limb [32]. This approach was validated through a randomized controlled trial showing better quadriceps symmetry and reduced compensatory activation patterns compared to control protocols.

However, limitations in these studies include small sample sizes, inconsistent use of motion analysis, and lack of long‐term follow‐up. Few protocols integrate real‐time biomechanical feedback or dynamic gait re‐training into standard care.

### 5.3 Integration of Wearable Sensors and AI for Rehabilitation Monitoring

Recent years have witnessed the rise of wearable inertial sensors (IMUs) and markerless motion systems that enable high‐frequency, out‐of‐lab monitoring of gait mechanics. For instance, the MoJoXlab system and related sensor‐based platforms capture critical features such as stance duration, tibial rotation, and impact loading with high fidelity [8, 21, 22].

These data streams, when combined with machine learning (ML) models, have enabled identification of key predictive gait features associated with delayed recovery or compensatory strategies [14, 15]. Explainable AI models further offer real‐time interpretability to clinicians, allowing adaptive modification of rehabilitation exercises. One study using AI‐assisted gait classification demonstrated high sensitivity in detecting non‐normalized gait during field‐based activities, surpassing clinician‐based observational scoring [15].

Such tools not only facilitate remote monitoring and early detection of aberrant mechanics but also support biofeedback‐based interventions, which may accelerate symmetry restoration.

### 5.4 Evidence‐Based Guidelines and Remaining Gaps

Consensus documents and systematic reviews have proposed updated ACLR rehabilitation guidelines, stressing the need for biomechanically validated progression criteria and limb symmetry thresholds rather than purely strength or hop metrics [18, 29]. Yet, the uptake of such evidence into clinical practice remains limited.

Several challenges persist:

- Lack of standardization in defining gait normalization across labs and devices
- Limited translation of research‐grade biomechanical tools into real‐world clinic settings
- Insufficient long‐term RCTs comparing wearable‐guided rehab to standard care

Despite these gaps, integration of gait biomechanics, wearable sensors, and AI‐supported analytics promises to enhance precision rehabilitation, reducing reinjury risk and optimizing functional outcomes.

## 6 Conclusion

ACLR serves as a cornerstone in restoring knee joint stability, yet persistent gait abnormalities and neuromuscular asymmetries underscore the complexity of achieving full functional recovery. This review delineates a temporal framework for postoperative gait analysis, from early deficits in loading and support mechanics to mid‐term adaptations in joint coordination and long‐term compensatory strategies that may predispose individuals to secondary injury. Incorporating wearable sensors, AI‐assisted analytics, and electromyographic monitoring enables a more precise and longitudinal understanding of gait biomechanics. Moreover, rehabilitation strategies supported by randomized controlled trials (RCTs) and meta‐analyses suggest that tailored, stage‐specific interventions, particularly those targeting neuromuscular symmetry, joint load modulation, and motor relearning, are critical to optimizing outcomes. Future directions should focus on the integration of real‐time feedback systems, remote gait monitoring platforms, and personalized machine learning models to advance individualized rehabilitation protocols. Ultimately, a multimodal and technology‐enhanced approach to gait recovery may redefine standards in ACLR rehabilitation and secondary prevention.

## Author Contributions

**TszLeung Yu:** conceptualization, data curation, formal analysis, investigation, methodology, visualization, writing – original draft, writing – review and editing. **HengYu Liao:** investigation. **ZhenQiang Huo:** investigation. **ZhiYu Huang:** supervision, writing – review and editing. **JieRuo Li:** conceptualization, supervision, writing – review and editing. **XinYu Fang:** conceptualization, supervision, funding acquisition, writing – review and editing. All authors read and approved the final manuscript and agree to be accountable for all aspects of the work.

## Funding

This work was supported by the National Natural Science Foundation of China (Grant No. 82571554).

## Conflicts of Interest

The authors declare no conflicts of interest.

## Data Availability

Data sharing not applicable to this article as no datasets were generated or analyzed during the current study.
